# Global incidence of incomplete surgical excision in adult patients with non-melanoma skin cancer: study protocol for a systematic review and meta-analysis of observational studies

**DOI:** 10.1186/s13643-020-01350-5

**Published:** 2020-04-17

**Authors:** Grant S. Nolan, Justin C. R. Wormald, Ailbhe L. Kiely, Joshua P. Totty, Abhilash Jain

**Affiliations:** 1 Division of Surgery and Interventional Science, University College London, Royal Free Hospital, Pond Street, London, NW3 2QG UK; 2 Whiston Hospital, St Helens and Knowsley Teaching Hospitals NHS Trust, Warrington Road, Prescot, Merseyside, L35 5DR UK; 3 Nuffield Department of Orthopaedics, Rheumatology and Musculoskeletal Sciences, University of Oxford, Roosevelt Drive, Oxford, OX3 7LD UK; 4 Stoke Mandeville Hospital, Buckinghamshire Healthcare NHS Trust, Mandeville Rd, Aylesbury, HP21 8AL UK; 5 Royal Stoke University Hospital, Newcastle Rd, Stoke-on-Trent, ST4 6QG UK; 6 Castle Hill Hospital, Hull University Teaching Hospitals, Castle Road, Cottingham, East Riding of Yorkshire HU16 5JQ UK

**Keywords:** Skin neoplasia, Carcinoma, Basal Cell, Carcinoma, Squamous Cell, Margins of excision, Systematic review

## Abstract

**Background:**

Non-melanoma skin cancer, which includes basal cell carcinoma and cutaneous squamous cell carcinoma, is the commonest malignancy worldwide. The mainstay of treatment is surgical excision. Despite this being an exceptionally common procedure, it is not known what the accepted standard is for incomplete excision. Multiple single-centre, regional and national studies have previously reported their incidence of incomplete excision in isolation. Furthermore, is it not known what effect potential risk factors such as the operating group, location of lesions, type of reconstruction, histological components or use of loupe magnification have on the incidence of incomplete excisions. The objective of this study will be to systematically evaluate observational data that present incidence of incomplete surgical excision amongst adult patients with non-melanoma skin cancer worldwide.

**Methods:**

We designed and registered a study protocol for a systematic review and meta-analysis of descriptive epidemiology data. A comprehensive literature search will be conducted (from January 2000 onwards) in MEDLINE, EMBASE, Scopus, CINAHL, EMCare and Cochrane Library. Grey literature will be identified through searching Open Grey, dissertation databases (e.g. Open Access Theses and Dissertations) and clinical trial registers (e.g. WHO ICTRP). Observational studies (cohort, cross-sectional, case series and clinical audits) reporting the incidence of incomplete surgical excision and conducted in adult patients with non-melanoma skin cancer will be included. The primary outcome will be the incidence of incomplete surgical excision (defined as residual tumour at either the peripheral or deep margin). Secondary outcomes will be risk factors that may affect incomplete excision (e.g. operating group, location of lesions, types of reconstruction, histological components). Data will not be extracted if the study uses other surgical techniques such as Mohs micrographic surgery, intra-operative frozen section, incision, shave or punch biopsies. Two investigators will independently screen all citations, full-text articles and abstract data. Potential conflicts will be resolved through discussion. No limitations will be imposed on publication status or language of publication. The study methodological quality (or bias) will be appraised using an appropriate tool. If feasible, we will conduct a random effect meta-analysis of observational data. Incidence estimates will be stratified according to cancer type (e.g. basal cell carcinoma vs squamous cell carcinoma) and operating group (e.g. dermatology, plastic surgery and general practice). Additional analyses will be conducted to explore the potential sources of heterogeneity (e.g. methodological quality, sample size).

**Discussion:**

This systematic review will summarise the best available evidence and definitively establish the incidence of incomplete surgical excision in non-melanoma skin cancer. It will determine if there is variation observed amongst different operating groups and provide some evidence for potential other factors causing this difference. This knowledge will provide a standard for future audits and will contribute to improving the treatment of non-melanoma skin cancer treatment.

**Systematic review registration:**

PROSPERO CRD42019157936

## Background

Non-melanoma skin cancer is an umbrella term which includes basal cell carcinoma (BCC) and cutaneous squamous cell carcinoma (SCC) as the most prevalent subtypes. They are the commonest cancers in the UK accounting for 20% of all new malignancies [1]. The UK incidence is 124–148 per 100,000 person years [2], and is projected to rise, likely due to increased reporting and historic exposure to ultraviolet radiation. By 2020, skin cancer (including melanoma) is estimated to cost the NHS over £180 million per annum [3].

Diagnosis is usually straightforward from clinical examination [4]; however, in the case of uncertainty, a small subset of lesions undergo incision or punch biopsy for definitive diagnosis.

The treatment of BCC and SCC may be through surgical and non-surgical methods. Surgical methods can be further divided into destructive techniques, such as cryotherapy or cautery, and non-destructive techniques i.e. surgical excision. Surgical excision (also termed simple excision, wide-local excision or non-micrographic excision) is where the skin cancer is excised with a ‘cuff’ of normal tissue. This aims to remove the macroscopic tumour along with any microscopic tumour deposits in the surrounding tissue. It is generally the mainstay of treatment for non-melanoma skin cancer in the UK, as it allows examination of the histological subtype and accurate assessment of margins. Excision confirms the complete removal of both macro and microscopic tumour. Clear margins are important as long-term outcomes are highly dependent upon achieving them; just 1% [5, 6] of BCC recur where margins are clear, compared to 31–41% recurrence where margins are involved [7, 8]. Recurrent lesions may require further surgery or increased surveillance where a ‘watch and wait’ approach is taken. Where lesions with incomplete margins do go on to be re-excised, only 45–55% [8, 9] reveal residual tumour cells. Incomplete excisions are a burden to both patients and healthcare systems, increasing costs and the morbidity of skin cancer care.

In the UK, excision of skin cancer is predominantly performed in secondary care [10], and there is joint guidance from the National Institute for Health and Care Excellence (NICE) and the British Association of Dermatologists (BAD) which includes ideal surgical margins [4, 11]. These guidelines use data from Mohs micrographic surgery to extrapolate the risk of incomplete excisions, stating that a 4–5-mm peripheral margin will achieve clear margins in approximately 95% of small, well-defined BCCs [4]. A similar approach, extrapolating SCC excision margins from Mohs micrographic surgery, forms the basis of UK SCC guidelines [12].

The figure of 5% incomplete excision with a 4–5-mm peripheral margin is therefore an estimation and not based on clinical evidence. Several previous large-scale studies such as two national audits of BCC and SCC excisions by UK dermatologists have reported an incidence of incomplete excision of between 2.3 and 3% [13, 14]. There have been no previous systematic reviews on this topic. It is common practice for dermatology and plastic surgery units to audit their own percentage of incomplete excisions and a global standard based on clinical data is required. This review is required to base this standard upon.

The objective of this study will be to systematically evaluate published observational studies that present incidence of incomplete surgical excision amongst adult patients with non-melanoma skin cancer worldwide.

## Methods

This protocol has been registered with PROSPERO international prospective register of systematic reviews (registration number CRD42019157936) and has been reported in accordance with the Preferred Reporting Items for Systematic Reviews and Meta-Analysis Protocols (PRISMA-P) 2015 statement [15]. The PRISMA-P checklist for this study is included in Additional file 1. The methodology of this review will be according to the Cochrane Handbook for Systematic Review of Interventions [16]. The final review will be reported following the PRISMA statement and the Meta-Analysis of Observational Studies in Epidemiology (MOOSE) guidelines [17].

### Information sources and search strategy

The primary source of literature will be a structured search of major electronic databases (from January 2000 onwards—considering skin cancer care has progressed over time and data more than 20 years old is likely not representative of current clinical practice): MEDLINE (Ovid), EMBASE (Ovid), Scopus (Elsevier), CINAHL (EBSCO), EMCare (OVID) and Cochrane Library.

The secondary source of potentially relevant material will be a search of the grey or difficult to locate literature, including Open Grey, dissertation databases (e.g. Open Access Theses and Dissertations) and clinical trial registers (e.g. World Health Organization International Clinical Trials Registry Platform). We will perform hand-searching of the reference lists of included studies, relevant reviews, national clinical practice guidelines or other relevant documents to identify cited articles not captured by electronic searches. Content experts and authors who are prolific in the field will be contacted. The literature searches will be designed and conducted by the review team which includes two experienced health information specialists. The search will be performed in English. Translations will be obtained for non-English articles. The search will include a broad range of terms and keywords related to non-melanoma skin cancer and epidemiological studies. The full search strategy used is provided in Additional file 2.

### Study selection

Study selection will be conducted in a two-stage process. The titles, and if required the abstracts, will initially be screened by two reviewers, using pre-specified screening criteria, for potential eligibility after excluding duplicate records. This process will be performed in Rayyann [18], which is a bespoke web and mobile app for systematic reviews. Relevant studies will then undergo full-text review by both reviewers. Translations will be obtained for non-English articles. Any discrepancies between reviewers will be resolved by discussion or by referral to a third reviewer. The search results, including abstracts, full-text articles and record of the reviewer’s decisions, including reasons for exclusion, will be recorded in a combination of Rayyan [18] and Microsoft Excel (Microsoft Corporation, 2018).

### Eligibility criteria

Studies will be selected according to the following criteria: participants, condition or outcome of interest and study design. No limitations will be imposed on publication status (unpublished studies will be eligible for inclusion) or language of publication. Data in the studies must have been collected after January 2000, considering skin cancer care has progressed over time and data more than 20 years old is likely not representative of current clinical practice. Therefore, studies reporting data which starts before January 2000, and finished after this date will be included but those studies reporting data solely from before January 2000 will be excluded.

### Participants/population

We will include studies involving adult population (≥ 18 years old) undergoing surgical excision of the two commonest forms of non-melanoma skin cancer (BCC and SCC).

BCC (diagnostic code C44.91) is a slow growing, locally aggressive tumour that arises from the basal layer of the epidermis on sun-exposed areas of the body [19]. Metastasis is extremely rare [20]. The clinical appearance of BCC is diverse including nodular, superficial, morphoeic and pigmented varieties amongst others. There are several common histological subtypes that include nodular, superficial, morphoeic and infiltrative, of which the latter two are particularly associated with aggressive tissue invasion and destruction, making these higher risk for incomplete excision [21].

Primary cutaneous SCC (diagnostic code C44.92) is a malignant tumour of keratinocytes which arises in the epidermis but shows histological evidence of dermal invasion [22]. It is locally invasive and has the potential to metastasises via lymphatic spread to other organs of the body.

Ultraviolet exposure causing sunburn in fair skinned individuals is the major risk factor for both BCC and SCC; however, SCC has several other risk factors including immunosuppression. SCC may also occur in chronic wounds, scars and old thermal burns [23, 24].

Surgical excision (also termed simple excision, wide-local excision or non-micrographic excision) is defined as an operative procedure where the skin cancer is excised with a ‘cuff’ of normal tissue. This may be undertaken under local, regional or general anaesthetic. Loupe magnification may be used by the operator; however, there should be no formal examination of the margins until the sample is received by the pathology laboratory. Studies reporting on Mohs micrographic surgery, or surgery including frozen section specimens will be excluded.

Only patients who have a confirmed histological diagnosis of BCC or SCC from surgical excision will be included. Those patients who have a clinical diagnosis of BCC or SCC that at histology is found to be an alternative diagnosis will be excluded. Studies reporting on SCC of the perineum and external genitalia (e.g. anal, vulvar and penile SCC) will not be included as the treatment of these conditions is specific to their region and these are not usually treated by dermatologists or plastic surgeons. Additionally, studies on metastatic SCC will be excluded as the deep margin clearance in this case becomes less relevant to clinical outcomes [11].

### Outcome measures

The primary outcome will be the incidence of incomplete surgical excision (defined as the residual tumour found either the peripheral or deep margin). ‘Closely’ or ‘near to’ excised lesions will be considered as fully excised in line with previous studies and recorded as such [25].

Secondary outcomes will be risk factors that may affect incomplete excision (e.g. operating group, location of lesions, types of reconstruction, histological components).

### Study design

Eligible studies will be observational studies (e.g. cohort, cross-sectional, case series and clinical audits) reporting incidence data using validated tools and conducted in a wide range of clinical settings (including data from administrative databases and registries). Cross-sectional studies will be the most appropriate study design to determine the incidence of incomplete surgical excision. For cohort studies, only the first phase (cross-sectional) data will be considered. We will exclude letters, reviews, case reports and case series with fewer than 50 patients. Studies using Mohs micrographic surgery or with intra-operative frozen section will be excluded, as will incision, shave or punch biopsies. No limitations will be imposed on publication status (unpublished studies will be eligible for inclusion), or language of publication.

### Setting

Studies performed in any clinical setting will be included.

### Data extraction

The data from all full-text articles included in the review will be independently retrieved by two reviewers using a standardised electronic data extraction form in Microsoft Excel (Microsoft Corporation, 2018). The following data will be extracted:

- Study characteristics
  - Authors, year of publication, journal, country, study design
  - Inclusion criteria, exclusion criteria
- Time period of data collection
- Patient demographics
  - Total number of patients
  - Number excluded
  - Number of males/females
  - Mean age + standard deviation
- Number of lesions excised (BCC, SCC)
  - Number lesions excluded
- Primary outcomes
  - Number of incomplete excision (BCC, SCC)
- Risk factors that may affect the incidence of incomplete excision
  - Operating practitioner (dermatology/plastic surgery/general practice/others)
  - Number of lesions on head and neck
  - Reconstructions (number undergoing direct closure, skin grafts, flaps, secondary intention, other)
  - Number with one or more high-risk histological components [11, 26] (excluding incomplete excision for SCC)
  - Use of loupe magnification

In the case of missing data, we will contact authors via email asking them to provide these details. As we do not expect authors of studies published more than 10 years ago to respond to inquiries, we will only contact authors of studies published from 2010 onwards, and only when contact details (email address) are provided in the publication. Research has previously shown that about 30% of trial authors are unreachable and 40% or more do not respond to emails, even after several reminders [27]; therefore, we have decided that if no reply is forthcoming, or the message cannot be delivered, we will not try to contact the authors again.

### Assessment of risk of bias of included studies

The risk of bias will be assessed at the individual study level by two review authors independently. Discrepancies will be resolved through discussion or referral to a third reviewer. The included studies will all be observational (e.g. cohort and case control studies) in nature and some will be uncontrolled (e.g. case series and clinical audits). These will be assessed using a risk of bias tool specifically designed for incidence studies [28] which was developed from a tool originally designed by Leboeuf-Yde and Lauritsen [29]. This comprises 10 signalling questions plus a summary assessment where questions 1 to 4 assess the external validity of the study (covering the domains of selection and non-response bias) and items 5 to 10 assess the internal validity (covering domains of measurement bias and bias related to analysis). Responses for individual items are either high or low risk of bias, where if there is insufficient data to decide the default is high risk of bias. The summary assessment evaluates the overall risk of study bias and is based on the rater’s subjective judgement given responses to the preceding 10 items which is based on the Grade of Recommendations, Assessment, Development and Evaluation (GRADE) and Cochrane approaches [16, 30] Response options for the summary assessment are low, moderate or high risk of bias. Signalling question 9 was not deemed to be appropriate to this systematic review and was therefore omitted.

A hypothetical clinical audit with a low risk of bias was pre-conceptualised and input into the risk of bias tool. This has been included as Additional file 3.

The results of the risk of bias tool will be used in a sensitivity analysis to ensure studies judged to be at ‘high’ risk of bias do not affect the robustness of our results in any subsequent meta-analysis.

### Data analysis and synthesis

To address the main review questions, data will be synthesised to establish the global incidence of incomplete surgical excision of BCC and SCC over the last 20 years. The data from each included study will be used to build evidence tables of an overall description of included studies for BCC and SCC separately. This will include study characteristics, context, participants, outcomes and findings. Crude incidence estimates (number of cases/sample size) will be presented along with 95% confidence intervals. If feasible and appropriate, incidence data from primary observational studies will be used to perform a meta-analysis of proportions with a random effects model [31]. This approach is appropriate given it is likely that the true incidence rate varies from study to study and these follow a normal distribution. This will allow us to estimate the pooled prevalence and its 95% confidence intervals using the random effects model with logit and back transformation for BCC and SCC separately. This will be presented as a forest plot.

To determine the extent of variation between selected studies, tests of heterogeneity will be performed. Inter-study heterogeneity will be assessed visually using the forest plot. Statistical heterogeneity will be quantified statistically using three tests. The *I*^2^ statistic will be used and the result will be interpreted using the definitions in the Cochrane Handbook for Systematic Reviews of Interventions [16]. Additionally, the *χ*^2^ and *τ*^2^ statistic will be used where a *p* value < 0.05 will be deemed as statistically significant for heterogeneity. Any sources of heterogeneity will be explored using subgroup analysis.

### Additional analysis

If sufficient studies are identified and data points are available, potential sources of heterogeneity will be investigated further by subgroup or meta-regression analyses. We plan to conduct analysis to establish the incidence of incomplete excision for each operating group (dermatology, plastic surgery and general practice) as well as the effect of the potential risk factors for incomplete excision (location of lesions, type of reconstruction, high-risk histological components.) A Wald-type test will be conducted to compare the summary effect sizes across subgroups: using either a Z-score or a Q-statistic, to determine whether or not two groups have significantly different outcomes [32].

In addition, we will explore incidence trends for each specialty and globally overall over time (with the year of publication as the explanatory variable) using random effects meta-regression models.

As the number of studies included in the review in anticipated to be large, we will undertake a sensitivity analysis to ensure the robustness of our results. We anticipate that the systematic review will identify many small studies which may be classified as at a higher risk of bias due to the limited reporting of them (e.g. through conference abstracts). We will therefore perform two sensitivity analyses in which abstracts and those studies judged to be at high risk of bias are excluded.

### Meta-basis

Small study effects (or publication bias across studies) will be assessed by inspecting a funnel plot for asymmetry if more than 10 studies are included.

## Discussion

Non-melanoma skin cancer is the commonest cancer worldwide of which surgical excision is the mainstay of treatment. This systematic review will aim to summarise the best available evidence and definitively establish the incidence of incomplete excision. This knowledge will establish a global standard of which future studies in this area can be compared to in order to improve skin cancer treatment worldwide. It may also elude to risk factors that influence the incidence, namely the operating practitioner, the location of lesions, the complexity of reconstructions undertaken and the presence of high-risk histological components. The knowledge derived from this review will provide a global standard for future audits and studies in this area. This standard will be based on recent clinical evidence, and crucially not an extrapolation from Mohs micrographic data which forms the basis of some national guidelines. Up-to-date epidemiological evidence about levels and trends in skin cancer care is essential information for effective national health policy and guidelines.

In this paper, we have presented a study protocol for a systematic review with meta-analysis. If any amendments or deviations from the protocol are required will be reported in the final manuscript. We plan to disseminate the results of this systematic review at national meetings/conferences of plastic surgeons and dermatologist and through publication.

At the study level there are several limitations that we anticipate. We expect the reported incidences of incomplete excisions to vary greatly from different studies. There are multiple reasons for this variation which may not be fully captured by our data collection; the size of lesions, seniority of surgeon and specific anatomical location are several factors which may affect the incomplete excision rate but are rarely reported in studies. Furthermore, the use of Mohs micrographic surgery by some operators such as dermatologists, may remove the most challenging and highest-risk tumours from their caseload which could reduce their incidence of incomplete excision.

Additionally, we expect a degree of reporting bias (through both non-publication of studies and selective reporting of outcomes) which may affect this systematic review. There is clearly a potential conflict of interest in terms of reporting one’s own incidence of incomplete excision which may affect funding from national bodies or cause patients to seek a different specialist in a private system. Authors will likely have less drive to publish data on a ‘bad’ audit cycle or may not report specific outcomes of interest like the incomplete excision rate which could bias the results. We hope to minimise this through contact authors for missing details and identifying publication bias using a funnel plot.

## Supplementary information

**Additional file 1.** PRISMA-P 2015 Checklist.

**Additional file 2.** Search Strategies.

**Additional file 3.** Risk of bias assessment in observational studies.

## Data Availability

Not applicable.

## Abbreviations

BAD: British Association of Dermatologists
BCC: Basal cell carcinoma
CINAHL: The Cumulative Index to Nursing and Allied Health Literature
EMBASE: Excerpta Medica Database
GRADE: Grade of Recommendations, Assessment, Development and Evaluation
MeSH: Medical Subject Headings
MOOSE: Meta-Analysis of Observational Studies in Epidemiology
NHS: National Health Service
NICE: National Institute for Health and Care Excellence
PRISMA-P: Preferred Reporting Items for Systematic Review and Meta-Analysis Protocols
RCTs: Randomised controlled trials
SCC: Cutaneous squamous cell carcinoma
WHO ICTRP: World Health Organization International Clinical Trials Registry Platform
UK: United Kingdom of Great Britain and Northern Ireland
