# Supplementary material for: Global incidence of incomplete surgical excision in adult patients with non-melanoma skin cancer: study protocol for a systematic review and meta-analysis of observational studies
Source: Syst Rev. 2020 Apr 17;9:83. doi: 10.1186/s13643-020-01350-5 (PMC7164252; doi:10.1186/s13643-020-01350-5)
Supplement: Supplementary file 2 — Additional file 2. Search Strategies. [file 13643_2020_1350_MOESM2_ESM.docx]

Additional file 2: Search Strategies

**Medline (EBSCO) –**

**Search Date 27/11/19**

incomplet* N3 excis* OR incomplet* N3 remov* OR inadequat* N3 excis* OR inadequat* N3 remov* OR incomplete N3 margin* OR inadequat* N3 margin* OR excis* N3 error* OR margin* N3 error* OR remov* N3 error* OR re-excis* OR "peripheral margin*" OR "positive margin*" OR excis* N3 margin* OR "lateral margin*" OR "deep margin*" OR "involv* margin*" OR error* N3 margin*

**AND**

"non-melanoma*" OR nonmelanoma OR "basal cell" OR "squamous cell" OR "rodent ulcer*" OR "non melanoma*"

OR MW squamous cell carcinoma OR MW carcinoma, basal cell OR MW carcinoma, squamous cell

**AND**

skin OR cutaneous OR dermatolo* OR dermis OR epidermis

OR MW skin

**Cinahl (EBSCO)**

**Search Date 27/11/19**

incomplet* N3 excis* OR incomplet* N3 remov* OR inadequat* N3 excis* OR inadequat* N3 remov* OR incomplete N3 margin* OR inadequat* N3 margin* OR excis* N3 error* OR margin* N3 error* OR remov* N3 error* OR re-excis* OR "peripheral margin*" OR "positive margin*" OR excis* N3 margin* OR "lateral margin*" OR "deep margin*" OR "involv* margin*" OR error* N3 margin*

**AND**

"non-melanoma*" OR nonmelanoma OR "basal cell" OR "squamous cell" OR "rodent ulcer*" OR "non melanoma*"

OR MW squamous cell carcinoma OR MW carcinoma, basal cell OR MW carcinoma, squamous cell

**AND**

skin OR cutaneous OR dermatolo* OR dermis OR epidermis

OR MW skin

**Embase (OVID)**

**Search Date 28/11/19**

incomplet* ADJ3 excis* OR incomplet* ADJ3 remov* OR inadequat* ADJ3 excis* OR inadequat* ADJ3 remov* OR incomplete ADJ3 margin* OR inadequat* ADJ3 margin* OR excis* ADJ3 error* OR margin* ADJ3 error* OR remov* ADJ3 error* OR re-excis* OR "peripheral margin*" OR "positive margin*" OR excis* ADJ3 margin* OR "lateral margin*" OR "deep margin*" OR "involv* margin*" OR error* ADJ3 margin*

**AND**

"non-melanoma*" OR nonmelanoma OR "basal cell" OR "squamous cell" OR "rodent ulcer*" OR "non melanoma*"

OR SH squamous cell carcinoma OR SH basal cell carcinoma OR SH non melanoma skin cancer

**AND**

skin OR cutaneous OR dermatolo* OR dermis OR epidermis

OR SH skin

**Emcare (OVID)**

**Search Date 28/11/19**

incomplet* ADJ3 excis* OR incomplet* ADJ3 remov* OR inadequat* ADJ3 excis* OR inadequat* ADJ3 remov* OR incomplete ADJ3 margin* OR inadequat* ADJ3 margin* OR excis* ADJ3 error* OR margin* ADJ3 error* OR remov* ADJ3 error* OR re-excis* OR "peripheral margin*" OR "positive margin*" OR excis* ADJ3 margin* OR "lateral margin*" OR "deep margin*" OR "involv* margin*" OR error* ADJ3 margin*

**AND**

"non-melanoma*" OR nonmelanoma OR "basal cell" OR "squamous cell" OR "rodent ulcer*" OR "non melanoma*"

OR SH squamous cell carcinoma OR SH basal cell carcinoma OR SH non melanoma skin cancer

**AND**

skin OR cutaneous OR dermatolo* OR dermis OR epidermis

OR SH skin

**Scopus (Elsevier)**

**Search Date 27/11/19**

incomplet* W/3 excis* OR incomplet* W/3 remov* OR inadequat* W/3 excis* OR inadequat* W/3 remov* OR incomplete W/3 margin* OR inadequat* W/3 margin* OR excis* W/3 error* OR margin* W/3 error* OR remov* W/3 error* OR re-excis* OR "peripheral margin*" OR "positive margin*" OR excis* W/3 margin* OR "lateral margin*" OR "deep margin*" OR "involv* margin*" OR error* W/3 margin*

**AND**

"non-melanoma*" OR nonmelanoma OR "basal cell" OR "squamous cell" OR "rodent ulcer*" OR "non melanoma*"

OR KW squamous cell carcinoma OR KW basal cell carcinoma OR KW non melanoma skin cancer

**AND**

skin OR cutaneous OR dermatolo* OR dermis OR epidermis

OR KW skin

**Cochrane**

**Search Date 27/11/19**

incomplet* NEAR/3 excis* OR incomplet* NEAR/3 remov* OR inadequat* NEAR/3 excis* OR inadequat* NEAR/3 remov* OR incomplete NEAR/3 margin* OR inadequat* NEAR/3 margin* OR excis* NEAR/3 error* OR margin* NEAR/3 error* OR remov* NEAR/3 error* OR re-excis* OR "peripheral margin*" OR "positive margin*" OR excis* NEAR/3 margin* OR "lateral margin*" OR "deep margin*" OR "involv* margin*" OR error* NEAR/3 margin*

**AND**

"non-melanoma*" OR nonmelanoma OR "basal cell" OR "squamous cell" OR "rodent ulcer*" OR "non melanoma*"

OR KW squamous cell carcinoma OR KW basal cell carcinoma OR KW non melanoma skin cancer

**AND**

skin OR cutaneous OR dermatolo* OR dermis OR epidermis

OR KW skin

**Open Grey**

**Search Date 27/11/19**

(remov* OR excis* OR margin* OR error*)

**AND**

(skin OR cutaneous OR dermatolo* OR dermis OR epidermis)

**AND**

("non-melanoma*" OR nonmelanoma OR "basal cell" OR "squamous cell" OR "rodent ulcer*" OR "non melanoma*")

**Open Access Theses and Dissertations**

**Search Date 27/11/19**

(remov* OR excis* OR margin* OR error*)

**AND**

(skin OR cutaneous OR dermatolo* OR dermis OR epidermis)

**AND**

("non-melanoma*" OR nonmelanoma OR "basal cell" OR "squamous cell" OR "rodent ulcer*" OR "non melanoma*")

**WHO ICTRP**

**Search Date 27/11/19**

excis* OR margin* **AND** cutaneous non-melanoma* OR cutaneous basal cell OR cutaneous squamous cell OR rodent ulcer* OR cutaneous non melanoma* OR cutaneous nonmelanoma* OR skin non-melanoma* OR skin basal cell OR skin squamous cell OR skin non melanoma* OR skin non melanoma*
