# Supplementary material for: Global incidence of incomplete surgical excision in adult patients with non-melanoma skin cancer: study protocol for a systematic review and meta-analysis of observational studies
Source: Syst Rev. 2020 Apr 17;9:83. doi: 10.1186/s13643-020-01350-5 (PMC7164252; doi:10.1186/s13643-020-01350-5)
Supplement: Supplementary file 3 — Additional file 3. Risk of bias assessment in observational studies. [file 13643_2020_1350_MOESM3_ESM.docx]

Additional file 3: Risk of bias assessment in observational studies ^1-4^

A hypothetical audit with a low risk of bias would provide the following answers:

|  | **Signalling question** | **Low risk example** |
| --- | --- | --- |
| **External validity** | 1. Was the study's target population a close representation of the national population in relation to relevant variables? | Assuming no age/gender predefined inclusion/exclusion criteria: |
|  | 2. Was the sampling frame a true or close representation of the target population? | All individuals with BCC/SCC included within the time frame of the study. |
|  | 3. Was some form of random selection used to select the sample, OR was a census undertaken? | Data collected on consecutive patients/excisions. |
|  | 4. Was the likelihood of nonresponse bias minimal? | Histological outcome on every patient with no missing data. |
| **Internal validity** | 5. Were data collected directly from the subjects (as opposed to a proxy)? | Outcome data extracted from original histology reports |
|  | 6. Was an acceptable case definition used in the study? | Incomplete excision defined as tumour at either peripheral or deep margin. Closely excised defined as clear.  Surgical excision was defined in an acceptable manner. Explicitly stated that incision, punch or Moh’s micrographic surgery was not used. |
|  | 7. Was the study instrument that measured the parameter of interest shown to have validity and reliability? | Incomplete excision was diagnosed independently by a qualified histopathologist in a recognised laboratory. |
|  | 8. Was the same mode of data collection used for all subjects? | Prospective data collection. |
|  | ~~9. Was the length of the shortest prevalence period for the parameter of interest appropriate?~~ | Not applicable. |
|  | 10. Were the numerator(s) and denominator(s) for the parameter of interest appropriate? | No errors in the reporting of numbers. |
| **Overall risk** | 11. Summary item on the overall risk of study bias | Low risk of bias: Further research is very unlikely to change our confidence in the estimate. |

References

1. Hoy D, Brooks P, Woolf A, et al. Assessing risk of bias in prevalence studies: modification of an existing tool and evidence of interrater agreement. J Clin Epidemiol 2012: 65: 934-9.

2. Leboeuf-Yde C, Lauritsen JM. The prevalence of low back pain in the literature. A structured review of 26 Nordic studies from 1954 to 1993. Spine (Phila Pa 1976) 1995: 20: 2112-8.

3. Higgins J, Green S. *Cochrane Handbook for Systematic Reviews of Interventions*. The Cochrane Collaboration, 2011.

4. Terracciano L, Brozek J, Compalati E, Schünemann H. GRADE system: new paradigm. Curr Opin Allergy Clin Immunol 2010: 10: 377-83.
